# Linking GPS Telemetry Surveys and Scat Analyses Helps Explain Variability in Black Bear Foraging Strategies

**DOI:** 10.1371/journal.pone.0129857

**Published:** 2015-07-01

**Authors:** Rémi Lesmerises, Lucie Rebouillat, Claude Dussault, Martin-Hugues St-Laurent

**Affiliations:** 1 Département de Biologie, Chimie et Géographie, Centre for Northern Studies, Université du Québec à Rimouski, Rimouski, Québec, Canada; 2 Département de Biologie, Chimie et Géographie, Université du Québec à Rimouski, Rimouski, Québec, Canada; 3 Direction de la gestion de la faune du Saguenay–Lac-Saint-Jean, Ministère des Forêts, de la Faune et des Parcs du Québec, Québec, Canada; 4 Département de Biologie, Chimie et Géographie, Centre for Northern Studies, Centre for Forest Research, Université du Québec à Rimouski, Rimouski, Québec, Canada; University of Tasmania, AUSTRALIA

## Abstract

Studying diet is fundamental to animal ecology and scat analysis, a widespread approach, is considered a reliable dietary proxy. Nonetheless, this method has weaknesses such as non-random sampling of habitats and individuals, inaccurate evaluation of excretion date, and lack of assessment of inter-individual dietary variability. We coupled GPS telemetry and scat analyses of black bears *Ursus americanus* Pallas to relate diet to individual characteristics and habitat use patterns while foraging. We captured 20 black bears (6 males and 14 females) and fitted them with GPS/Argos collars. We then surveyed GPS locations shortly after individual bear visits and collected 139 feces in 71 different locations. Fecal content (relative dry matter biomass of ingested items) was subsequently linked to individual characteristics (sex, age, reproductive status) and to habitats visited during foraging bouts using Brownian bridges based on GPS locations prior to feces excretion. At the population level, diet composition was similar to what was previously described in studies on black bears. However, our individual-based method allowed us to highlight different intra-population patterns, showing that sex and female reproductive status had significant influence on individual diet. For example, in the same habitats, females with cubs did not use the same food sources as lone bears. Linking fecal content (i.e., food sources) to habitat previously visited by different individuals, we demonstrated a potential differential use of similar habitats dependent on individual characteristics. Females with cubs-of-the-year tended to use old forest clearcuts (6–20 years old) to feed on bunchberry, whereas females with yearling foraged for blueberry and lone bears for ants. Coupling GPS telemetry and scat analyses allows for efficient detection of inter-individual or inter-group variations in foraging strategies and of linkages between previous habitat use and food consumption, even for cryptic species. This approach could have interesting ecological implications, such as supporting the identification of habitats types abundant in important food sources for endangered species targeted by conservation measures or for management actions for depredating animals.

## Introduction

Scat analyses are among the most intuitive tools used to describe animal diet and are simple, affordable and non-invasive [1–3]. Food remains are visually identified and their contribution to the total individual intake is estimated based on volume, weight, or occurrence in scat, and potentially corrected for their digestibility [4,5]. Many individuals can be sampled over long time periods without the negative impacts associated with the capture (e.g., tissue collection for stable isotope analyses) or the death of the animal (e.g., stomach content or gastrointestinal tract analyses; [6–8]).

Nevertheless, scat analyses have pitfalls and weaknesses such as challenges and biases associated with the identification of remains [9]. Moreover, although scat age has been estimated based on its appearance, climatic conditions (e.g., exposure to wind and sun) can alter scat, resulting in potentially skewed estimation of the seasonal diet phenology. Effectiveness of random sampling efforts could also influence results with variation associated with habitat structure and composition [10]. Indeed, scats are typically only collected through direct observation or detection by trained dogs. This could result in oversampling one individual or group of individuals [11], especially if they use open areas where feces are easier to locate or found in habitat types increasing dog efficiency [12]. This bias could ultimately weaken applicability of the results to the entire population. Removal of old feces from survey transects in a stratified sampling design could alleviate these problems by increasing accuracy of deposit date evaluation [11,13] while sampling all available habitats. However, implementing such an approach is challenging when studying sparsely distributed species, as low scat densities can impede the collection of large sample sizes. Another major weakness of traditional scat analyses is accurately linking individuals to scats collected [14]. Genotyping intestine epithelial cells that stick to the scat [3,15] or feeding marked animals with dyed foods [16] are among emerging approaches to solve this problem. These methods still remain limited because genotyping is only possible on fresh scats [15] and fed animals are susceptible to have disturbed foraging behavior while dye provides limited periods of scat identification efficiency [16].

GPS technology allows telemetry devices to transfer GPS locations via satellite (e.g., Argos or Iridium) or cellular (e.g., Lotek WildCell) link [17]. Researchers can then access the exact location visited by an animal, or a group of animals, in order to collect feces (as proposed by [18], but see also [19,20]). Furthermore, as devices can be programmed to record locations at any time interval, collecting information on habitat used at a fine spatiotemporal scale by collared individuals while they ingest food items is now feasible with improved accuracy (up to a few meters, depending on the terrain characteristics; [21]). Such an approach provides new opportunities for the study of the foraging ecology of cryptic species [16,18]. Coupling animal movements and habitat use with diet therefore provides a comprehensive method to detail animal foraging strategies. Feces content and life history traits of white-tailed deer (*Odocoileus virginianus* Zimmermann) were recently related by joining GPS/Iridium telemetry collars with biomarking of feces with food dyes [16]. The authors showed that it was possible to find tracks of individual deer in snow (as corroborated by dyed feces), to collect feces and to characterize foraging behavior based on comparison between food availability along tracks and random transects beside (15–30 m) tracks. Their sample size was unfortunately too low to draw strong conclusions on life history traits, yet they still demonstrated the applicability of the method in winter when snow cover allows individual path tracking.

The aim of this study was to relate habitat use patterns to feces content of black bear in the boreal forest of Québec, Canada. We captured bears, fitted them with GPS/Argos collars, and recorded individual characteristics such as sex, reproductive status, weight, body condition and age. We hypothesized that black bears concentrated their foraging activities in specific habitat types to collect certain food items. Specifically, we predicted that regenerating clearcuts would be used for forage bouts due to their availability and abundance of consumable food items such as blueberry (i.e., *Vaccinium angustifolium* A. or *Vaccinium myrtilloides* M.), ants, grass, and young leaves [22]. We also hypothesized that individual characteristics influenced both bear diet and foraging habitat. We predicted that abundant and protein-rich food (e.g., colonial ants) would be consumed by females with cubs due to the high energetic requirements of cub rearing. We further predicted that foraging would take place in habitats providing good cover to protect their offspring from predators and large males [23–27]. In contrast, we expected lone males and females to use open habitats that provide a high diversity and abundance of berries in order to quickly develop their fat reserves [22, 28].

## Methods

### Ethics Statement

In Canada, black bears are not considered as a species at risk according to the Committee on the Status of Endangered Species in Canada (COSEWIC). We thus captured, collared and released 21 individuals in strict accordance with the recommendations of the Canadian Council on Animal Care. Both captures and manipulations of study animals were approved by the Animal Welfare Committee of the Ministère des Forêts, de la Faune et des Parcs du Québec (hereafter referred to as MFFP; certificates #CPA-FAUNE 2011–30 and 2012–17). Captures were conducted on public lands, under the supervision of the Québec government (i.e., MFFP), so no specific permissions were required.

### Study area

The study area was located north of Saguenay (Québec, Canada) and covered approximately 6300 km², centered on Portneuf Lake (48°42’–49°17’N, 70°03’–70°42’W). Forests are transitional between the spruce–moss domain and the balsam fir *Abies balsamea* (Linnaeus) Mill.–white birch *Betula papyrifera* Marshall domain. Historical logging activities resulted in the harvest of ~35% of the forest area. Dense stands of deciduous trees, including willow *Salix* spp. Linnaeus and trembling aspen *Populus tremuloides* Mill., regenerate after logging. There is a high diversity of berry producing shrubs, the most common being blueberry, serviceberry *Amelanchier* Mill. spp., bunchberry *Cornus Canadensis* Linnaeus, skunk currant *Ribes glandulosum* Grauer, raspberry *Rubus ideaus* Linnaeus, wild and bristly sarsaparilla *Aralia nudicaulis* Linnaeus and *Aralia hispida* Vent., and chokecherry *Prunus virginiana* Linnaeus. Animal prey items available to black bear in the study area include beaver *Castor canadensis* Kuhl, moose *Alces americanus* Clinton, caribou *Rangifer tarandus caribou* Gmelin calves, snowshoe hare *Lepus americanus* Erxleben, ruffed grouse *Bonasa umbellus* Linnaeus, spruce grouse *Falcipennis canadensis* Linnaeus, as well as Canada goose *Branta canadensis* Linnaeus, which often nest in the area. Anthropogenic infrastructure is abundant and evenly distributed with a cabins density of 0.32 cabin/km^2^ and a forest road density of 1.8 km/km^2^. The mean annual temperature ranges between -2.5 and 0°C and annual precipitation fluctuates between 1000 and 1300 mm, of which 30 to 35% falls as snow [29]. The elevation ranges between 300 and 800 m with low rolling hills.

### Telemetry survey and sampling protocol

Black bears were captured in June and July of 2011 and 2012 using a padded foot snare or a culvert trap, then immobilized with a Telazol-Ketamin-Xylazine (5:4:1) mix. Adults (females > 54 kg and males > 68 kg, based on data from previous captures in the same area) were equipped with a GPS/Argos collar (TGW-4583H-2, Telonics, AZ, USA) programmed to attempt recording a location every two hours. Individual weight, length, premolar teeth (for age determination), and presence of young (visible cub, lactating cues, or information collected during den visits in winter of 2012 and 2013) were collected to build an individual set of intrinsic characteristics. Collars were programmed to send GPS locations from the 7 previous days once a week via Argos satellites at a rate of four collars per day. This schedule allowed us to investigate known locations of four different bears daily while ensuring that surveyed locations were not too old. We randomly selected two or four locations per bear each week to search for feces, depending on the week; because collars sometimes failed to connect with the Argos satellites, we temporarily increased sampling efforts on individuals that connected successfully. Feces found at each location (within a 10-m radius) were collected in a plastic bag and frozen until lab analysis. When a collared female was with her cubs, we distinguished adult and cub feces by their size and diameter. Cub feces were only used for general comparison purpose. Because black bear are primarily solitary or with cubs, except during the reproduction season (June 10^th^ to July 9^th^), and then only for few days [30], we assume that collected feces came from collared bears.

### Laboratory analyses

Frozen feces were warmed at room temperature for half a day before analyses and then gently mixed until homogeneous. A subsample of ~100 g was weighed and then washed through sieves (1-mm, 0.5-mm and 0.1-mm meshes) and the proportion of total remains present in the 1-mm and 0.5-mm sieves was estimated. The 0.1-mm sieve was used only to capture small and rare items that were not retained in the 0.5-mm sieve, or to help in fragment identification. Items were identified to the species level for ingested plants and mammals, while all bird species (mainly waterfowls, passerines and grouses species) were grouped together. Insects were classified as ants (adults and larvae) or yellow-jackets and wasps *Vespidae* spp. Anthropogenic food included pieces of plastic bag, corn, hen feathers, and other elements usually not found in nature. The detritus category included all non-food items, such as woody debris eaten while consuming ants, conifer needles, moss, small rocks, and other debris eaten by bears. Remains of each sieve were spread on hundred-checkered plates and proportions of each item category in the total volume (i.e., the number of 1-% squares filled compared to other remains) was assessed. The total contribution of each item or group of items in a particular scat was corrected according to their contribution in each sieve and the sieve proportion of the total remains retained. To account for differences in digestibility of food sources, we used correction factors, developed for black bear [31] and grizzly bear (*Ursus arctos horribilis* Ord), a closely related species [4]. These factors are based on the percent contribution of each item, yielding percent dry matter biomass ingested. Population means, by season and by item, were therefore calculated and presented with their standard deviation.

### Habitat use analyses

Previous studies estimated the average transit time of food items in the digestive tracts of black bear and brown bear (*Ursus arctos* Linnaeus), a closely related species, to range from ~ 4 to 16 hours [18,32,33]. We used the twelve locations previously recorded before the feces collection sites to characterize habitat use patterns 24 hours prior to the estimated feces deposition time. This allowed the inclusion of potential variability in gastrointestinal transit time depending on food type. The best time interval was subsequently assessed statistically (see statistical analyses section). We accounted for habitats used between GPS locations by considering habitat features contained within an ellipse encompassing two consecutive points. To do so, we used the package {adehabitatHR} [34] in R 2.15.3 [35] and performed Brownian bridges [36] using only two consecutive locations at a time. Different parameters had to be set *a priori* to control ellipse extent and shape. The first parameter (sig1) is related to animal speed and path tortuosity, and represents the probability that the animal diverges from the direct path (Euclidian distance), considering the distance and the time between successive locations. We selected this parameter with the *liker* function in {adehabitatHR} [36] using the annual individual location dataset to obtain an individual parameter value (mean = 4.98, min = 3.01, max = 6.76). The second parameter (sig2) represents the standard deviation of the distance from recorded GPS location to real animal location. As we filtered locations based on positional dilution of precision (PDOP < 10, SE of location error = 0.15, [37]), we set sig2 at 5; following *a priori* tests, we noted that this parameter had little influence on Brownian bridge shape and dimension unless it varied greatly (i.e., over what is commonly observed in GPS location precision standard deviation). The output was a raster with individual pixel values representing different probabilities of use by the bear between the two successive GPS locations. We transformed rasters into smoothed polygons using the *getverticeshr* function by including only pixels with a probability of use > 75% (as calculated by Brownian bridges) to obtain ellipses (see S1 Fig).

We used 1: 20,000 land cover maps provided by the MFFP, which are updated each year with new natural and anthropogenic disturbance polygons (e.g., forest fires, cutblocks, windthrows). Minimum mapping unit size was 4 ha for forested polygons and 2 ha for nonforested areas (e.g., water bodies). We classified forest stands into categories (Lake, Swamp, Conifer, Cut (0–5), Cut (6–20), Regeneration, Open; see Table 1 for description) relevant for bear ecology based on studies of their habitat selection in Québec [22,38]. Finally, the proportion of each habitat type and the density of anthropogenic structures (Table 1) were calculated within each ellipse using ArcGIS 10.0 [39].

**Table 1:** Description of variables and associated measurement units.

Variable	Description or scientific name	Unit	Variable	Description or scientific name	Unit
Secondary road	Forest road with low to high traffic	Km/km^2^	Ants	*Formicidae spp*.	% in feces
Closed road	Forest road with no traffic	km/km^2^	Poplar	*Populus tremuloides*	% in feces
River	Permanent running water	km/km^2^	Willow	*Salix spp*.	% in feces
Lake	Pond and lake	% in ellipses	Grass	*Carex spp; Juncus spp*.*; other graminoids*	% in feces
Swamp	Open wetland	% in ellipses	Mayflower berry	*Cornus canadensis*	% in feces
Conifer	Mature coniferous forest (>50 years old)	% in ellipses	Raspberry	*Rubus ideaus*	% in feces
Cut (0–5)	Forest clearcut (0–5 years old)	% in ellipses	Sarsaparilla	*Aralia hispida*	% in feces
Cut (6–20)	Forest clearcut (6–20 years old)	% in ellipses	Smilacina	*Smilacina trifolia*	% in feces
Regeneration	Old disturbance (20–40 years old)	% in ellipses	Creeping snowberry	*Gaultheria hispidula*	% in feces
Open	Non regenerated disturbance (> 20 years old)	% in ellipses	Blueberry	*Vaccinum myrtilloides; V*. *angustifolium*	% in feces
Beaver	*Castor canadensis*	% in feces			
Hare	*Lepus americanus*	% in feces			

### Statistical analyses

We frequently collected multiple feces (~45% of sites with > 1 scat, $\bar{x}$ = 1.80, SD = 1.24) at a single GPS location, especially at bear resting sites (i.e., where bears stay for 4 to 8 h). Ignoring the exact moment each feces was deposited can bias our analyses, but we took that into account by selecting the first location recorded in a 20-m buffer around the feces location (i.e., considering a ~10-m precision of GPS location) as the departure point for a 24-h backward ellipses delineation. These ellipses were used to characterize the habitat where bears potentially fed instead of where they rested. As we did not have information on the exact time each feces was dropped, we analyzed feces separately and then calculated the mean food items dry matter biomass in feces for each site.

We used the constrained correspondence analysis (CCA, library {vegan} [40]) to relate fecal content to individual characteristics and habitat used during foraging. To account for repeated measures on individuals (i.e., sites), we included individual as strata in ANOVAs determining axis significance. We applied the same procedure for the method *envfit* in library {vegan}, a permutation test (n = 999) performed to establish length, direction and significance of environmental vectors. This allowed the permutation of observations within the specified stratum in order to control for variance induced by intra-individual variation and unequal sampling.

We first related feces content to individual characteristics (i.e., sex, age, reproductive status, weight and body condition index) and grouped individuals based on shared traits. We then compared feces content of individuals sharing similar diets, as defined by the first CCA, with habitat use while foraging. We previously defined the time interval, among different possibilities in the 24 h range (S1 Table), which best explained the feces content. This was done using the proportion of variance explained by the CCA comparing the matrix of visited habitats (i.e., the proportion of cover type and density of roads and rivers in selected ellipses) with the matrix of food items identified in the feces of all individuals. We previously removed food items found in less than three feces to account for only relevant food sources. We also removed variables that were correlated (*r* > 0.6) and made sure that the variance inflation factor remained < 10 [41].

A population-level diet was also estimated for adult and for cubs by pooling all sites (mean dry mass of ingested food items ± SD) and separating them by season (spring; May 15^th^–June 14^th^, summer; June 15^th^–July 31^st^ and fall; August 1^st^–September 14^th^). Seasons were based on a sharp and clear shift in diet observed in our samples and to local plant phenology (unpublished data). We did not split diets by season for the individual-level analyses because of limited sample size.

All statistical analyses were carried out using R 2.15.3 and R 3.1.2 [35]. Data are available from the Dryad Digital Repository: http://dx.doi.org/10.5061/dryad.75r70 [42].

## Results

In 2012, we monitored 20 bears (6 males; 6 females with cubs-of-the-year, 6 females with yearlings, and 2 females alone, see Table 2) from May 15^th^ to September 15^th^. The GPS fix rate for the sampling period was 87.4%. We surveyed 374 GPS locations between 1 and 13 days after bears were located ($\bar{x}$ = 5.58, SD = 2.22). A total of 120 adult feces were found at 71 different GPS locations ($\bar{x}$ = 3.55 sites by bear, SD = 1.88, details in Table 2) and 30 cub feces in 13 different sites ($\bar{x}$ = 2.31, SD = 2.22). The time interval that best explained relationships between previously visited habitat and feces content was 10 to 16 h before excretion (see S1 Table), a result in accordance with previously estimated gastrointestinal transit times [18,32,33].

**Table 2:** Distribution of sample sizes among groups.

Group of individuals	Bear (*n*)	Sites				
		*n*	Mean	SD	Min	Max
Females with yearlings	6	17	2.8	1.8	1	5
Females with cubs	6	26	4.3	1.5	2	6
Lone females	2	2	1.0	0.0	1	1
Males	6	26	4.3	1.8	3	7
Total	20	71	3.6	1.9	1	7

**Fig 1:**
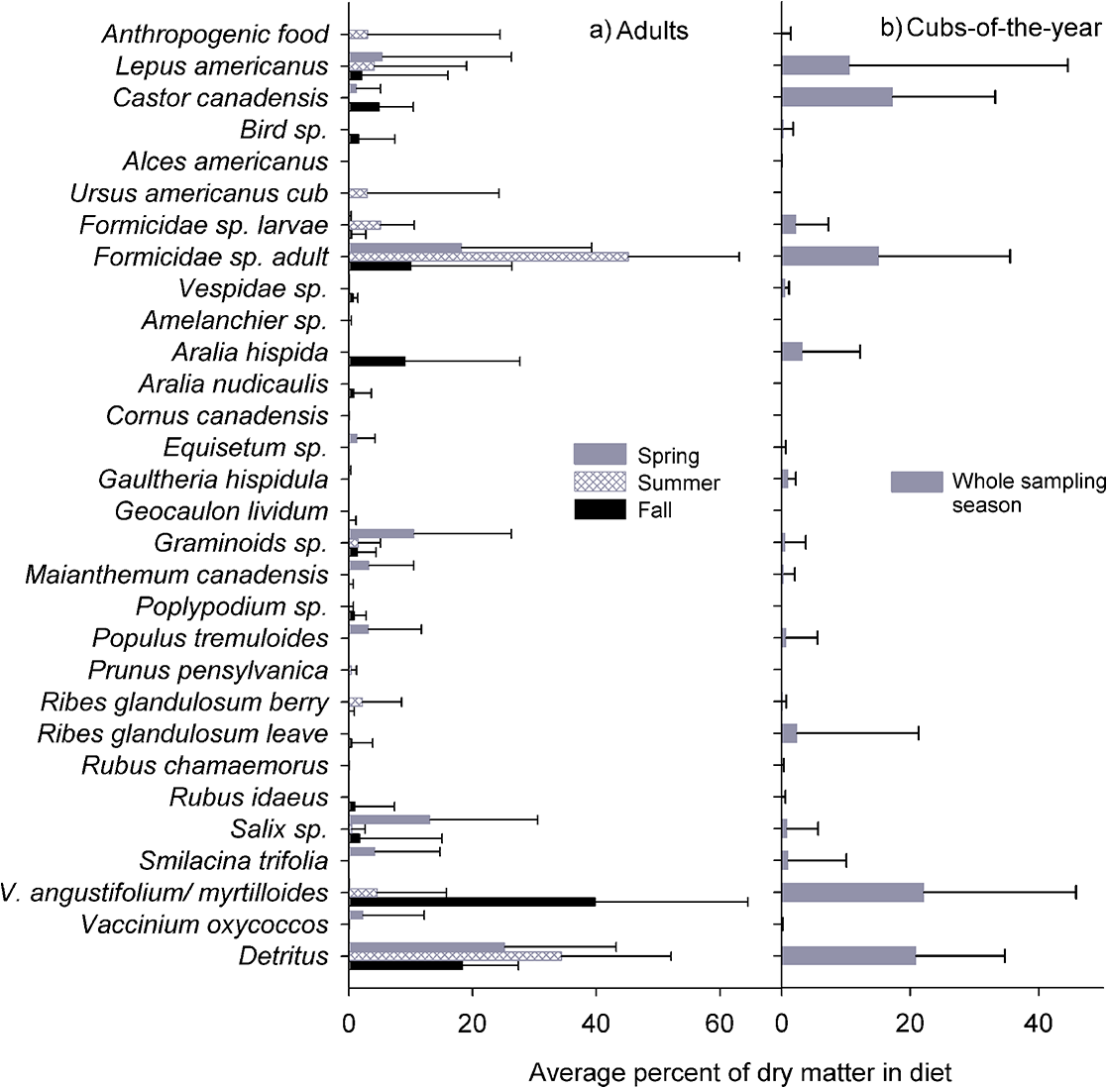
Corrected proportion of dry matter ingested of food items found in feces. a) Adults feces (*n* = 120 feces/71 sites) for spring (*n* = 47 feces/24 sites; May 15^th^–June 14^th^), summer (*n* = 32 feces/20 sites; June 15^th^–July 31^st^) and fall (*n* = 41 feces/27 sites August 1^st^–September 14^th^). b) Cub-of-the-year feces (*n* = 30 feces/13 sites) for the whole sampling season (May 15^th^–September 14^th^). Error bars represent the standard deviation.

Description of feces content at the population level varied seasonally with grasses, ants, snowshoe hares, and willow leaves being more abundant in spring (May 1^st^ to June 14^th^) (Fig 1A). Overwintered cranberries (*Vaccinium oxycoccos* Gray) and smilacina berries (*Smilacina trifolia* Linnaeus) were only found in feces excreted during spring, as well as other plant species that are edible during their first phenological stages (e.g., young leaves of deciduous trees). During summer (June 15^th^ to July 30^th^), bears had the least diverse diet of the entire sampled active season (May to September), relying almost exclusively on ants, especially species that dwelled in coarse woody debris. Ant larvae are known to occur in bear feces during that season, following their availability in ant colonies [43]. There was a positive relationship between the proportion of woody detritus and the proportion of ants in feces as most non-food items were pieces of wood eaten while ingesting ants. Interestingly, we noted evidence of cannibalism, as we found cub remains (i.e., claw, skin and fur) in a scat of a large, old male. Anthropogenic food was observed during the last days of the baiting season for bear hunting (May 15^th^ to June 30^th^). Berries were the main food source during the fall season but protein rich items such as ants, birds (i.e., *B*. *canadensis*, unidentified waterfowl and passerines), and beavers either increased or were still included in the diet. Cubs (Fig 1B) seemed to mainly eat highly digestible food items such as mammalian prey, ants and berries instead of leaves. This observation is however based on a limited sample size (n = 30 feces in 13 sites) for the whole sampling season.

**Fig 2:**
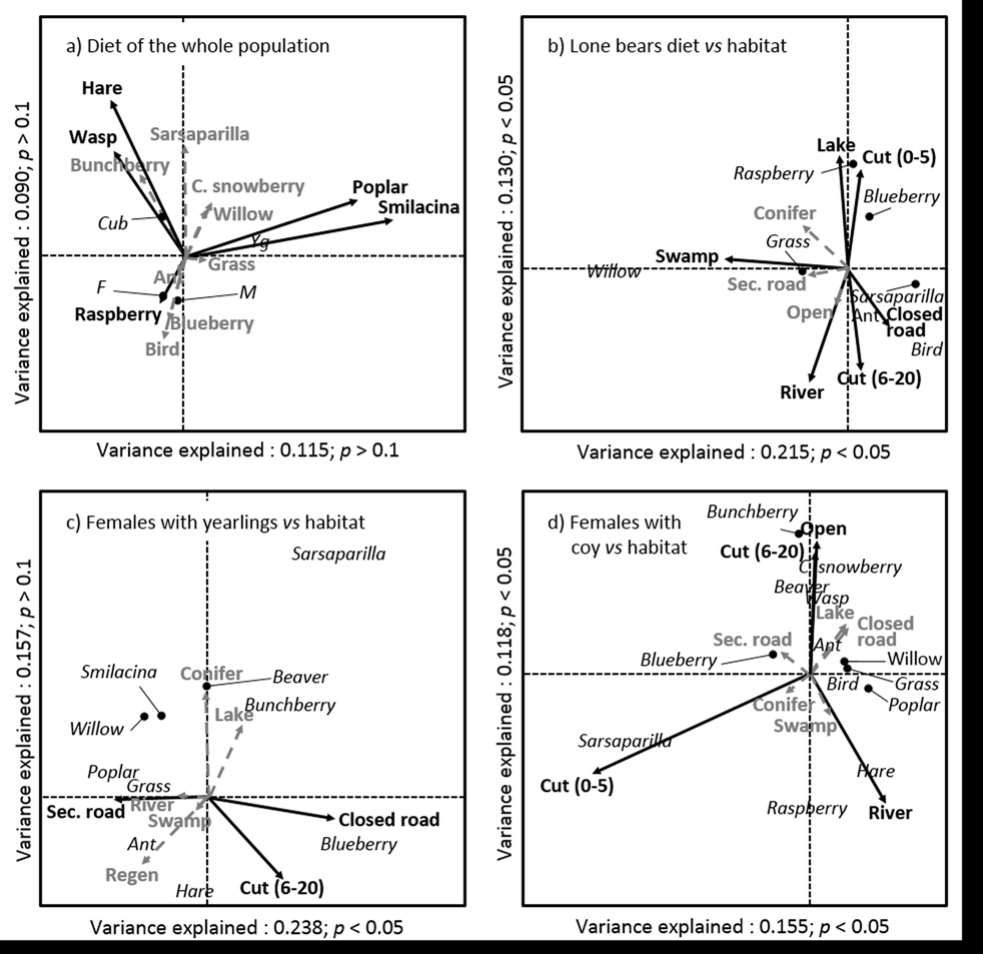
Graphical representations of Constrained Correspondence Analysis (CCA). Black arrows represent significant (*p* < 0.05) food item variables correlated with individual characteristics (in italics, panel *a*), and black arrows represent habitat variables correlated with the different food items (in italics, panel *b*,*c d*). Dashed grey arrows refer to variables that were not significantly correlated. In panel *a* (*n* = 28 feces of 6 different bears), body condition index and age are not shown to lighten the graphic and ease its interpretation as they were not significantly correlated with specific food items. Other panels represent relationships between habitat types and food items (in italics) by group of individuals sharing similar diets, as shown in panel *a*. Panel *b* for lone bears (*n* = 28 feces of 6 different bears), *c* for females with yearlings (*n* = 17 feces of 6 different bears) and *d* for females with cubs-of-the-year (*n* = 26 of 6 different bears).

The proportion of variance explained by the first two axes of the CCA between feces content and individual characteristics was 20.5%, although the ANOVA on axes yielded no significant results (Fig 2A). However, permutation test on vectors yielded five significant parameters (Fig 2A). Feces of females with cub-of-the-year appeared to be associated with animal prey, especially hares and *Vespidae* sp., when compared with lone bears of both sexes, while feces of females with yearlings were asociated with poplar and smilacina. The other intrinsic characteristics (i.e., age, body condition index, and weight) did not relate to any particular diet. Bears with similar diets were then grouped (i.e., females with cubs, females with yearling and lone bears of both sexes) and thereafter fecal contents were related to habitat use while foraging.

The first two axes of CCA between habitat in ellipses and feces content for each dietary group was always significant (except for the second axis of Fig 2C, ANOVA, *F = 2*.*90*, *p* > 0.1). The variance explained by the first two axes varied but was always higher than ~ 27% (lone males and females: 34.5%; females with yearlings: 39.5%; females with cubs-of-the-year: 27.3%; Fig 2B–2D), which indicates that diet was related to habitat use patterns. It further suggests that these relationships differ between sex and reproductive status. The analysis conducted for lone bears (Fig 2B) suggested that presence of blueberries and raspberries in scats was related to lake proportion in ellipses, which is probably indicative of the importance of lake shores and young cutovers (Cut 0–5). Ants and birds were found mainly in the feces of bears whose ellipses contained older cuts (Cut 6–20) and dense ‘Closed roads’, while feces of bears that visited ‘Swamp’ were more likely to include grasses and willows. The main food sources of females with yearling cubs was also correlated to the presence of older cuts (Cut 6–20) and ‘Closed roads’ in ellipses (Fig 2C). Females with yearling cubs and lone bears focused on blueberry and ants, respectively, when moving through the same habitat types. Our analyses also revealed the importance of roads for females with yearlings. Indeed, secondary road density in ellipses was associated with consumption of poplar and grasses (Fig 2C), important food sources during the spring period. Finally, we found four habitat types that can explain feces content for females with cubs-of-the-year (Fig 2D): ‘Cut 0–5’ was correlated to sarsaparilla while ‘Cuts 6–20’ and open unregenerated stands were correlated with bunchberry, wasp, beaver, creeping snowberry and ant (Fig 2D). Hare, found to be a food source favored by females with cubs (Fig 2A) was associated with high river density.

## Discussion

We linked GPS technology and scat analyses to relate feces to individual traits and to describe fine-scale habitat use patterns during foraging bouts by a free-ranging, cryptic mammal. We followed black bears throughout their active season and linked individual characteristics (i.e., sex, age, reproductive status, and body condition) to diet. Moreover, we related groups of individuals sharing similar diets to habitat use patterns during expected foraging bouts. This method represents an interesting approach to study animal diet in the wild as it foregoes the need to characterize ingested food items through direct observation [14]. Indeed, such characterisation can be a laborious task for cryptic species such as black bears and is often associated with numerous potential biases, notably the influence of the observer on animal foraging behavior and the difficulty of seeing and identifying all ingested food items [13,44].

### Diet at the population level

Traditionally, fecal analyses were used to determine the average diet composition of a population without assigning feces to a unique individual, except in studies using DNA analyses on epithelial cells (e.g. [3,15]) and recent studies using GPS locations (e.g. [20,45,46]). To make our results comparable to previous studies we performed our analyses at both the population and individual/group levels.

At the population level, seasonal diet estimates were similar to observations made in the boreal forest by other research teams (e.g. [22,46]), especially for plants and insects. However, the diversity and importance of mammal prey species we documented seemed relatively uncommon for black bears. For example, we found beaver remains in feces during spring and fall (2.7% and 6.8% of the dry matter biomass found in feces, respectively), although beaver has seldom been identified as a primary prey item in terms of both the availability and ingestion of prey items (but see [47] for an isolated bear population on an island in Lake Superior). Similarly, snowshoe hare, particularly abundant in our study area during the survey, was a common food item in bear diet, especially in spring and summer (8.1% and 7.7% of the dry matter biomass found in feces, respectively). This could be explained by the high availability of leverets during these periods [48]. The importance of hare also exceeded what has typically been observed in North American black bear diets (e.g. [13,22]). In contrast, moose and caribou calves were almost absent from our samples even though these ungulate prey are common in the study area. Moose calf hairs were only found in one cub scat and we cannot ascertain whether the calf was preyed on or scavanged. The diet of cubs, which includes more digestible food items than the diet of adults, could be an artifact of the onset of weaning after the period of leaves palatability [30,31].

### Individual variation in diet

We found variation in diet composition between individuals and these differences were related to sex and reproductive status, but not to age or body condition, as shown by the CCA. The abundance of the main food sources (i.e., ants, blueberry, grass, and willow) in scats did not differ between bears. Some food items were nonetheless found in higher abundance in feces of bears of same sex and reproductive status. For example, hare and vespidae were consumed more frequently by females with cubs-of-the-year, whereas young poplar leaves and smilacina berries of the previous year were more often observed in feces of females accompanied by yearlings. We believe that such contrasted patterns could be related to different habitat use and physiological requirements, although we did not measure individual energetic balance. Indeed, lactating females have high energetic demands related to cub nursing, especially in proteins [26]. Small mammal prey species could therefore be of great importance, especially prey living in closed and dense forest stands such as hares [49]. Incidentally, these habitats are also selected by females with cubs in our study area, probably for protection against predators and infanticidal males [25].

### Linking fecal content to habitat use

As some groups of individuals seemed to share similar and distinct diets, we looked further to see if these patterns were translated to relationships between ingested food items and habitats used during foraging bouts. Bear foraging activity was probably not limited to the habitats visited between successive locations, and bears were not eating only during the time interval considered for a foraging bout (10–16 hours). Nonetheless, it remains highly likely that the majority of food items found in feces originated from those habitats and that the Brownian bridge ellipses encompassed most of the habitats that were really used by bears, regardless of the inherent inclusion of unused habitats. This is supported by multiple significant relationships that we highlighted between food sources and particular habitat types using a limited number of replicates and a relatively short time interval (10–16 h).

Our results suggest that the link between fecal contents and habitat use varied between groups, with consistent behavioral differences between sex and reproductive status. While lone bears of both sexes used closed roads and old cuts (6–20 years old) to feed on ants and birds, females with yearlings and females with cubs selected similar habitat types and focused their foraging activity on blueberry and on bunchberry, beaver, and creeping snowberry, respectively. This is a possible example of differential use of similar habitat types. Although difficult to ascertain and strongly dependant on sample size, habitat use delineation, and food availability (not measured in this study), such patterns could result from intraspecific competition for food [30], discrepancy in nutritional needs [26], or displacement of females with young by lone bears to prevent aggression and cannibalism on offsprings [25]. Temporal segregation could also explain such differences. Indeed, blueberries, an abundant fall food source, were consumed by females with yearlings in these habitats. Lone bears, on the other hand, fed on ants, which is mostly a summer resource (Fig 1). Although both groups foraged in these habitat types, it seems that they did not use them during the same season and sought different food sources.

Analyses of habitat use and diet content for females with cubs-of-the-year showed lower explained variation than for other groups (Fig 2B, 2C and 2D), a counterintuitive result, especially for a group that undergoes strong selective pressure [26,50]. This could highlight the inconsistensies in foraging behavior patterns within this group of females. Although they share similar behavioral constraints (e.g., cubs protection and nursing [23–27]), the availability of habitat types within their seasonal home range may vary, increase the intra-group variance and mask common strategies between females.

### Methodological advantages and cautionary comments

We used a traditional approach by analyzing scat contents and went a step further by linking diet composition to timing of feeding and fine-scale habitat use patterns obtained from GPS/Argos telemetry. Nonetheless, decreasing location intervals and use of inertial navigation systems coupled with GPS [17] could fill the remaining gap between fixes and yield a continuous path for a better habitat use delineation. We chose the interval that best explained the relationship between feces content and habitat used, in accordance with literature on black and brown bears. However, feeding captive black bears with similar diet than their wild counterparts [18] could enhance the reliability of our data by identifying specific and more precise time intervals (although captivity could also influence transit time). Moreover, increasing sample size would be a critical feature, both in terms of individuals considered and of feces collected by individual, especially if the objective is to study inter-individual variation in foraging strategies.

We recognize that other techniques (e.g., isotopic ratios, GPS collars with video camera) could partially overcome these limitations, but note that considerable uncertainty still surrounds these methods. Scat DNA analyses are alternate ways to relate feces to a specific animal and assess diet diversity. The former implies a recovery of the feces shortly after excretion to prevent DNA damage of intestine epithelial cells found on the surface of feces [51] and yields little information on individual characteristics besides sex. The latter (diet analysis based on DNA metabarcoding) is a reliable but expensive way to detect all ingested food items, provided that appropriate DNA markers are used. The performance of these markers to assess the relative importance of each item in biomass ingested is however still debated, especially for diverse diets [52].

## Conclusions and Management Implications

We were able to identify black bear behaviors that were almost impossible to otherwise uncover for this cryptic species, such as differential use of similar habitat by distinct groups of bears and concentration of foraging activities in group-specific habitats. Being able to document different foraging strategies within a population could have strong implications for management. For example, knowing what a critical segment of an endangered population eats and, more importantly, where it feeds, could enhance recovery actions and identify habitats that require specific conservation measures. Some individuals might not concentrate their activity in habitat recognized to provide abundant food sources [22,38]. It could then be important to identify limiting factors that foster this behavior (e.g., predator avoidance, anthropogenic disturbances) to limit their impact and allow individuals to use optimal habitats for foraging. In contrast, identifying individuals exhibiting problematic behaviors (e.g., habituated bear feeding on anthropogenic food near human facilities) could help focus control actions and support in understanding individual characteristics that may promote detrimental or potentially dangerous behaviors.

## Supporting Information

S1 Fig Schematic representation of a 24-hr displacement of a black bear before the feces excretion point. The 75% ellipses generated using Brownian bridges between each pair of consecutive GPS locations (registered at a 2h interval) represent the area potentially visited by the individual, allowing for nonlinear paths between locations. The shaded ellipses represent the 6–12h before excretion time interval considered in subsequent analyses. Excretion point is represented here by a star.
(TIF)

S1 Table Proportion of variance explained by the two first components of the CCA. This table refers to the comparison of matrices of visited habitat with the related feces’ food items. Best time interval (in hours) is represented in bold characters.
(DOCX)
