# Supplementary figures and images for: Linking GPS Telemetry Surveys and Scat Analyses Helps Explain Variability in Black Bear Foraging Strategies
Source: PLoS One. 2015 Jul 1;10(7):e0129857. doi: 10.1371/journal.pone.0129857 (PMC4489386; doi:10.1371/journal.pone.0129857)

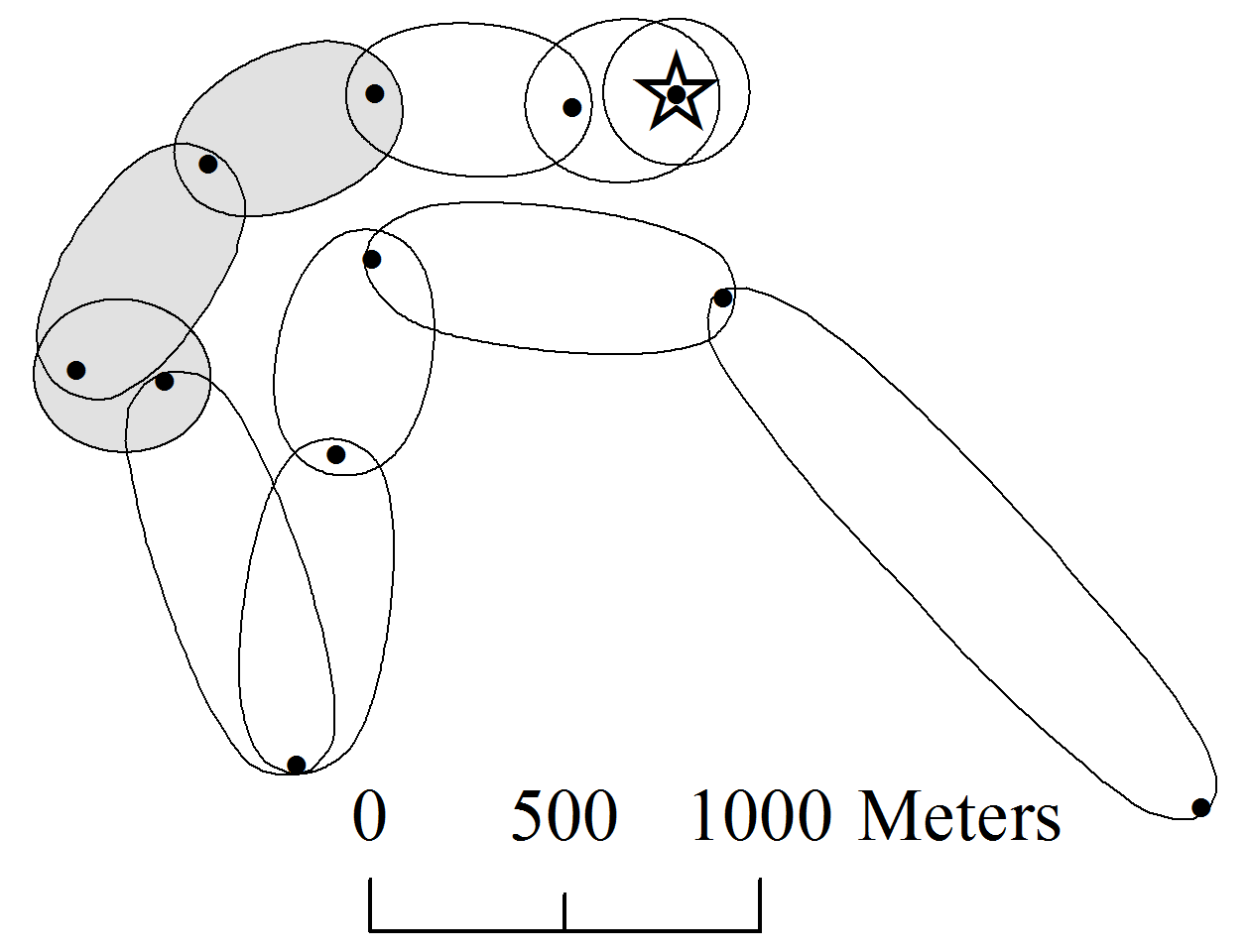

Supplement: S1 Fig — The 75% ellipses generated using Brownian bridges between each pair of consecutive GPS locations (registered at a 2h interval) represent the area potentially visited by the individual, allowing for nonlinear paths between locations. The shaded ellipses represent the 6–12h before excretion time interval considered in subsequent analyses. Excretion point is represented here by a star. (TIF) [file pone.0129857.s001.tif]
