# Supplementary material for: Linking GPS Telemetry Surveys and Scat Analyses Helps Explain Variability in Black Bear Foraging Strategies
Source: PLoS One. 2015 Jul 1;10(7):e0129857. doi: 10.1371/journal.pone.0129857 (PMC4489386; doi:10.1371/journal.pone.0129857)
Supplement: S1 Table — This table refers to the comparison of matrices of visited habitat with the related feces’ food items. Best time interval (in hours) is represented in bold characters. (DOCX) [file pone.0129857.s002.docx]

**S1 Table.** **Proportion of variance explained by the two first components of the CCA.** This table refers to the comparison of matrices of visited habitat with the related feces’ food items. Best time interval (in hours) is represented in bold characters.

| Time interval | Total variation explained |
| --- | --- |
| 0 to 4 | 0.190 |
| 0 to 6 | 0.185 |
| 2 to 6 | 0.185 |
| 2 to 8 | 0.185 |
| 4 to 8 | 0.183 |
| 4 to 10 | 0.187 |
| 6 to 10 | 0.193 |
| 6 to 12 | 0.194 |
| 8 to 12 | 0.199 |
| 8 to 14 | 0.219 |
| 10 to 14 | 0.235 |
| **10 to 16** | **0.255** |
| 12 to 16 | 0.251 |
| 12 to 18 | 0.243 |
| 14 to 18 | 0.233 |
| 14 to 20 | 0.223 |
| 16 to 20 | 0.216 |
| 16 to 22 | 0.209 |
| 18 to 22 | 0.205 |
